# Dataset on potential biochemical activities of Cylo phe-Ala-Asp-Gly-based compounds as Caspase 1 inhibitor

**DOI:** 10.1016/j.dib.2025.111503

**Published:** 2025-03-21

**Authors:** Faith Eniola Olujinmi, Chijioke John Ajaelu, Sunday Adewale Akintelu, Abel Kolawole Oyebamiji

**Affiliations:** a Industrial Chemistry Programme, Bowen University, PMB 284, Iwo, Osun, Nigeria; b Department of Industrial Chemistry, University of Ilesa, Ilesa, Osun State, Nigeria; c Good Health and Wellbeing Research Clusters (SDG 03), University of Ilesa, Ilesa, Nigeria

**Keywords:** Caspase 1, Peptides, Inflammation, *In silico*, Pharmacokinetics

## Abstract

In this work, seven Cylo phe-Ala-Asp-Gly-based Compounds were optimized using Spartan’14 software. Series of software used in this work were Spartan 14, molecular operating environment (MOE), padel, ADMET. The optimized compounds were docked against human Caspase 1 (6F6R) so as to observe their inhibiting ability. The calculated descriptors for individual compounds were reported and described. Also, the docked compound 2 (2-((2S,8S,11S)-11-([1,1′-biphenyl]-4-ylmethyl)-8-benzyl-3,6,9,12-tetraoxo-1,4,7,10-tetraazacyclododecan-2-yl) acetic acid) proved to be more efficient in inhibiting human caspase 1 than other compounds under investigation. The pharmacokinetic evaluation of compound with highest binding affinity and reference compound were executed and reported*.*

Specifications Table

**Table:** 

Subject	*Computational Chemistry*.
Specific subject area	*Drug Design and Analysis*
Type of data	Raw Data, Figure, Chart, Scheme, Table, ADMET, Graph
Data collection	Seven Cylo phe-Ala-Asp-Gly-based Compounds were modelled and subjected to density functional theory calculation via B3LYP (6-31+G*) for theoretical calculations using Spartan ‘14. The compounds were calculated in three phases (gas, ethanol and water) and the descriptors were presented accordingly. The docking calculation was accomplished using induced fit method for molecular docking investigation. The pharmacokinetic evaluation of the compound with best binding affinity was executed using ADMETSar 2.0 and all the results were reported appropriately.
Data source location	Computational Chemistry Research Laboratory, Department of Chemistry and Industrial Chemistry, BOWEN University, PMB 284, Iwo, Osun State, Nigeria.
Data accessibility	***Please note:****All raw data referred to in this article must be made publicly available in a data repository prior to publication. Please indicate here where your data are hosted (the URL must be working at the time of submission and editors and reviewers must have anonymous access to the repository*): Repository name: Mendeley Data Data identification number: *doi:*10.17632/hn36p8mp4r.1 Direct URL to data: https://data.mendeley.com/datasets/hn36p8mp4r/1 Instructions for accessing these data: The data can be accessed using the above URL.
Related research article	*None.*

## 1 Value of the Data

• The molecular descriptors obtained from the optimized compound in the three different phases will provide information to researchers (most especially, organic Chemist) on the effect of solvent and most suitable solvent for the compounds.
• The calculated binding affinity for the optimized compound against (PDB ID: 6F6R) Caspase-1 will help scientists (especially, pharmaceutical scientists/ medicinal Chemist) to identify compounds that possess the best configuration with utmost efficiency.
• The 2- dimensional structures of the compounds under investigation will reveal to researchers the nature of the derivatives attached to the parent compound.
• The distance between every atom in the compounds and the amino acid residues will expose to scientists the level of spontaneity of the examined peptide-based compound and receptor.
• The pharmacokinetic analysis will provide insight into how the compound with the best binding affinity is absorbed, digested, metabolized and excreted in the body and also reveal the toxicity level.
• The molecular dynamic simulation will help researchers to confirm the ligand with utmost potential anti- Caspase 1 properties.

## 2 Background

The objectives of this work are:

➢ To evaluate solvent effect on the chemical activity of the examined Cylo phe-Ala-Asp-Gly-based Compounds
➢ To observe the interaction between the compound and the amino acid residues in the active site of Caspase-1 (PDB ID: 6F6R)
➢ To reveal the pharmacokinetics activity of the compound with the best binding affinity*.*
➢ To identify the molecule with highest potential capacity to inhibit Caspase 1 which will thereby help in predicting libraries of efficient compounds

## 3 Data Description

Table 1 reveals the seven compounds which are all derivatives of the parent compound: Cylo phe-Ala-Asp-Gly, the IUPAC nomenclature and the two-dimensional structure of the compounds investigated in this work are displayed in Table 1.

**Table 1:** Two-dimensional structure of the examined peptide based compounds.

Table 2 exposes the descriptors obtained for the investigated compound. The highest occupied molecular orbital (HOMO), lowest unoccupied molecular orbital (LUMO) and the energy gap in all three phases are detailed in Table 2.The location of the orbital energy levels HOMO-LUMO for the seven compounds being examined are listed in Table 3. HOMO-LUMO was obtained for all the optimised compounds in the vacuum, water and ethanol using dot format for the three phases (Table 4, Table 5).

**Table 2:** Calculated DESCRIPTORS for compounds optimized in vacuum, water and ethanol.

	Vacuum			Water			Ethanol		
	HOMO	LUMO	EG	HOMO	LUMO	EG	HOMO	LUMO	EG
1	-7.05	-1.34	-5.71	-6.91	-1.37	-5.54	-6.82	-1.42	-5.4
2	-6.71	-1.57	-5.14	-6.8	-1.36	-5.44	-6.77	-1.29	-5.48
3	-6.75	-1.52	-5.23	-6.55	-1.44	-5.11	-6.56	-1.38	-5.18
4	-6.72	-1.52	-5.2	-6.17	-1.4	-4.77	-6.16	-1.34	-4.82
5	-7.1	-3.78	-3.32	-6.91	-4.22	-2.69	-6.84	-3.89	-2.95
6	-6.68	-1.57	-5.11	-6.76	-1.37	-5.39	-6.74	-1.3	-5.44
7	-6.75	-1.56	-5.19	-6.45	-1.44	-5.01	-6.46	-1.38	-5.08

**Table 3:** Predicted orbital energy levels for compounds optimized in vacuum.

**Table 4:** Predicted orbital energy levels for compounds optimized in water.

Table 6 reveals the binding affinity of the Cylo phe-Ala-Asp-Gly compounds against the human caspase 1 enzyme (PDB ID: 6F6R). The binding affinity is -6.5045414 kcal/mol for compound 1, -6.9591408 kcal/mol for compound 2, -7.248467 kcal/mol for compound 3, -6.9861174 kcal/mol compound 4, -7.0307384 kcal/mol for compound 5 -6.8699188 for compound 6, -7.4287872 kcal/mol and the reference drug -7.4287872 kcal/mol with which all the compound was compared.

**Fig. 1:**
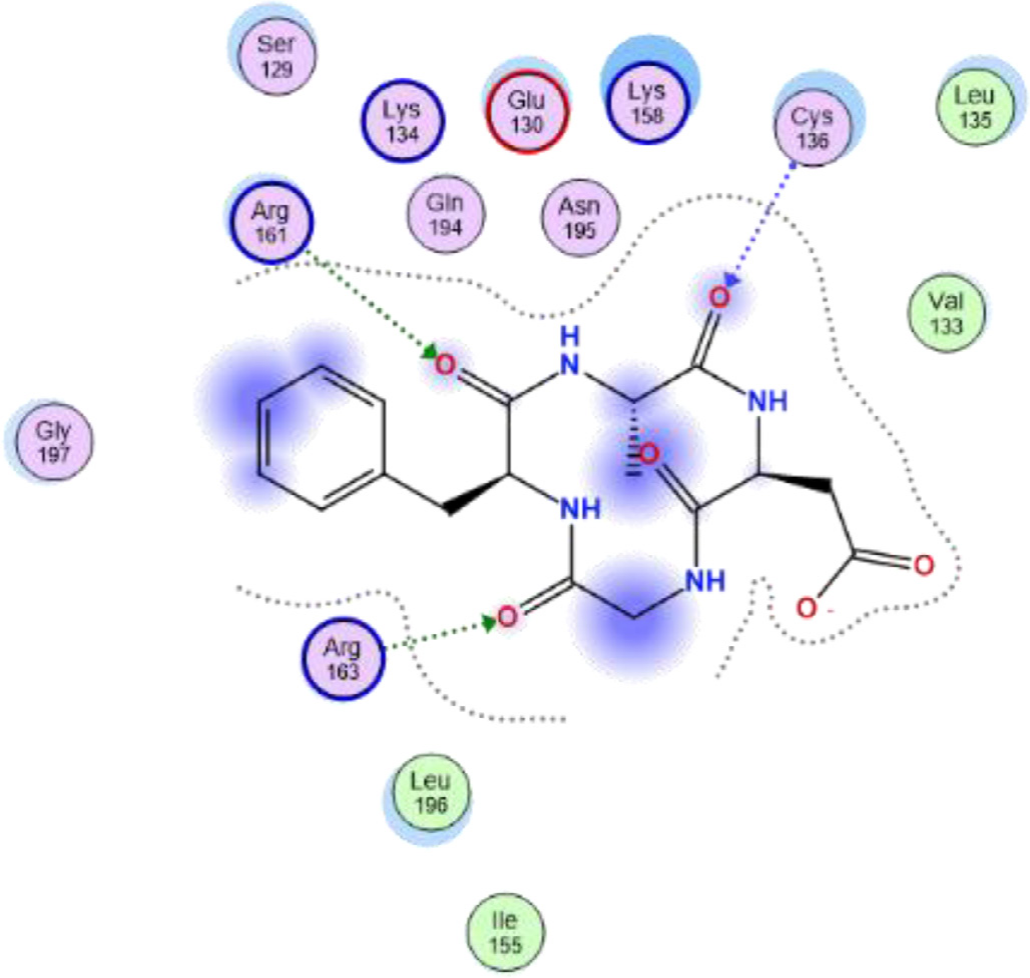
2-dimensional structure for docked Compound 1 against human Caspase 1 [pdb id: 6F6R].

**Fig. 2:**
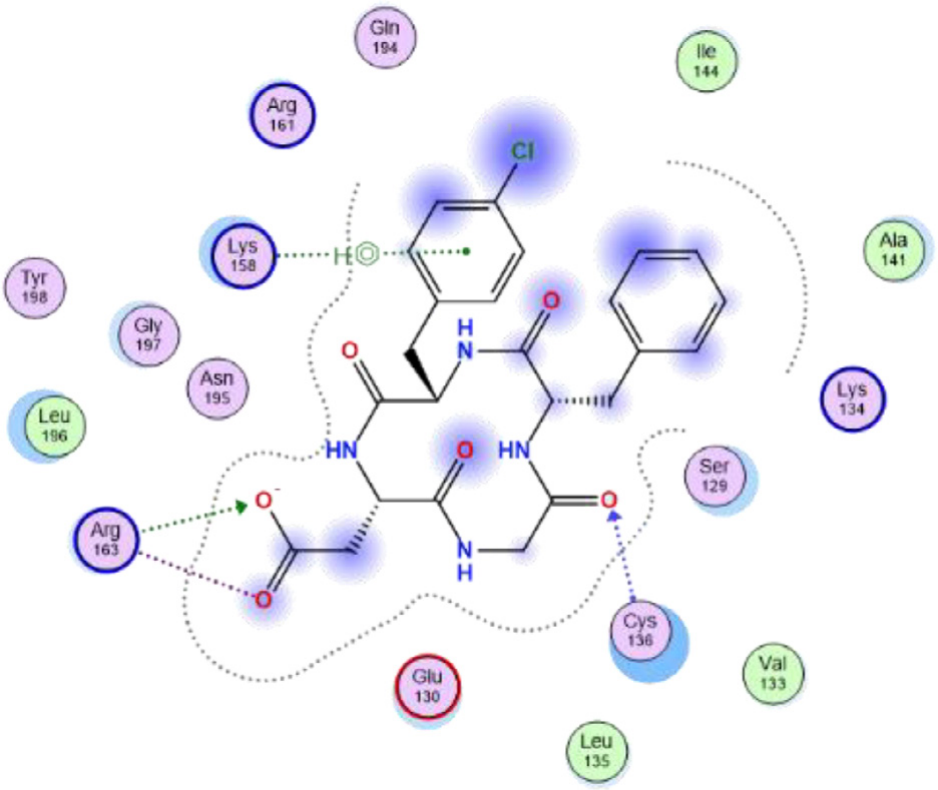
2-dimensional structure for docked Compound 2 against human Caspase 1 [pdb id: 6F6R].

**Fig. 3:**
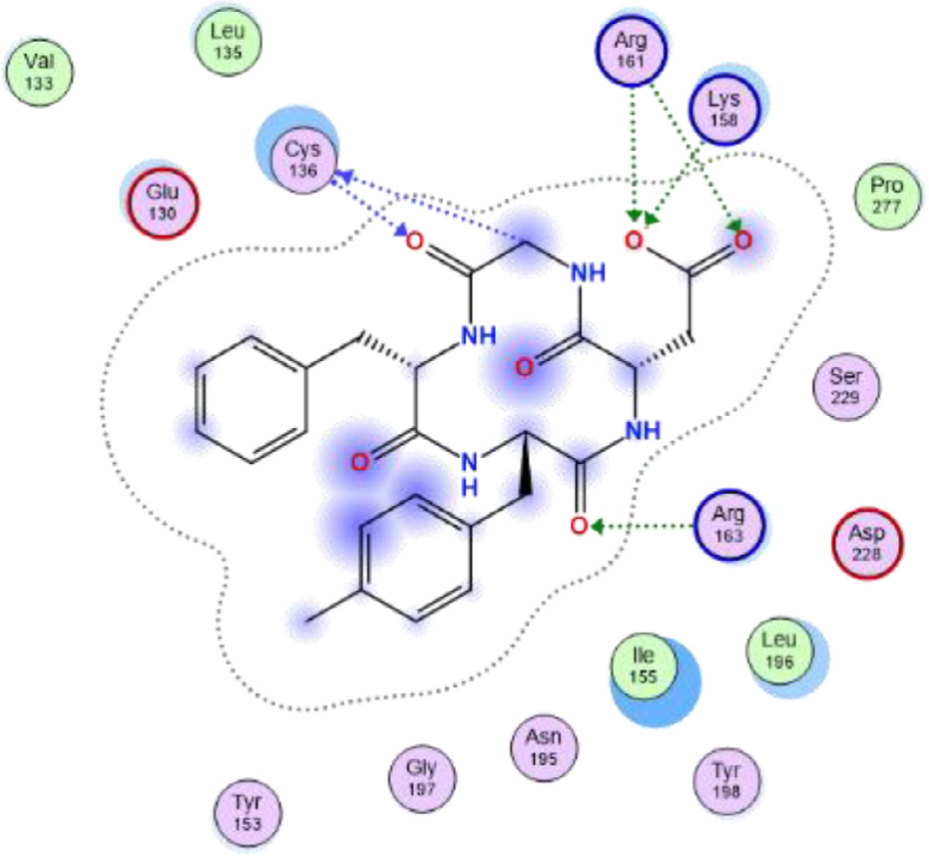
2-dimensional structure for docked Compound 3 against human Caspase 1 [pdb id: 6F6R].

**Fig. 4:**
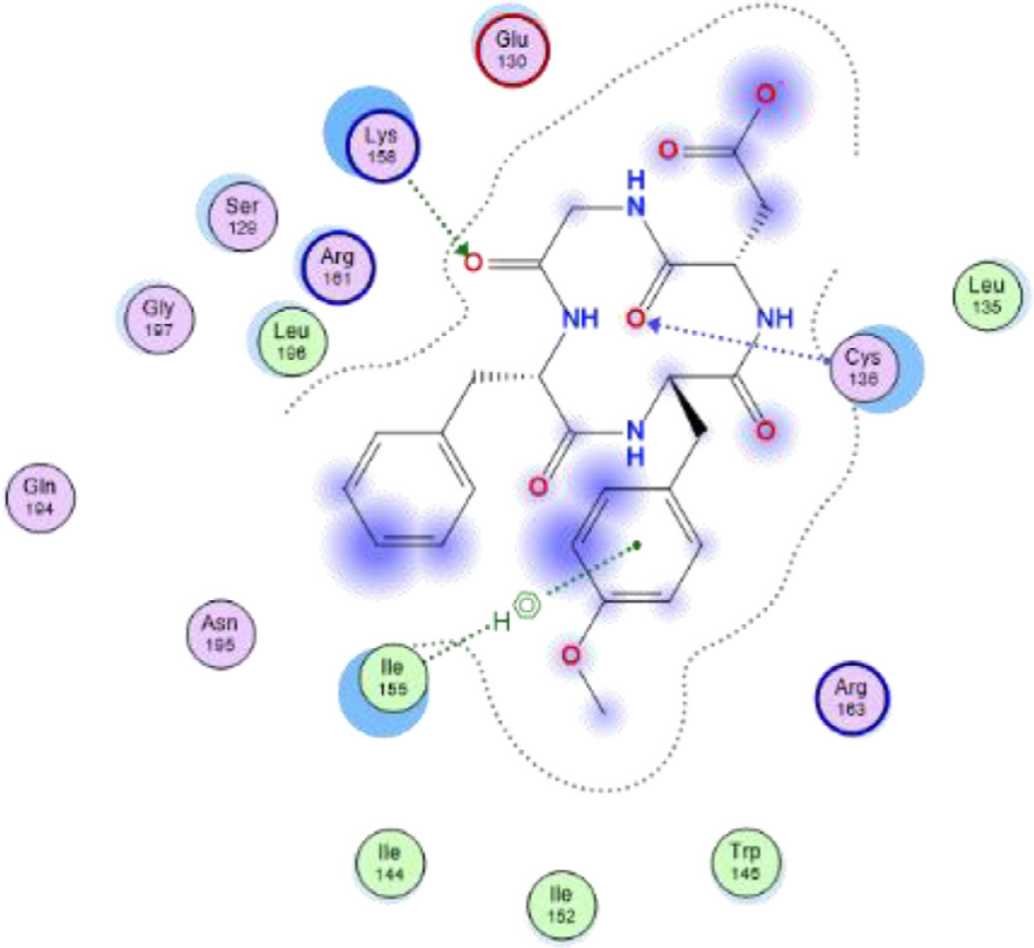
2-dimensional structure for docked Compound 4 against human Caspase 1 [pdb id: 6F6R].

**Fig. 5:**
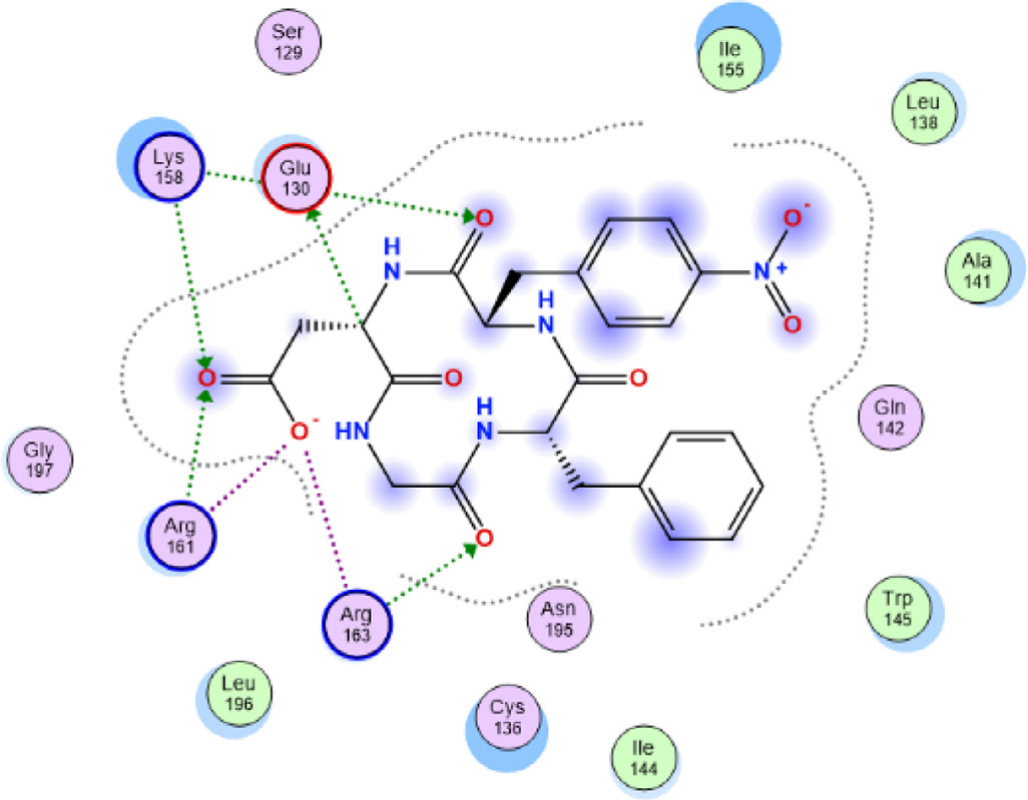
2-dimensional structure for docked Compound 5 against human Caspase 1 [pdb id: 6F6R].

**Fig. 6:**
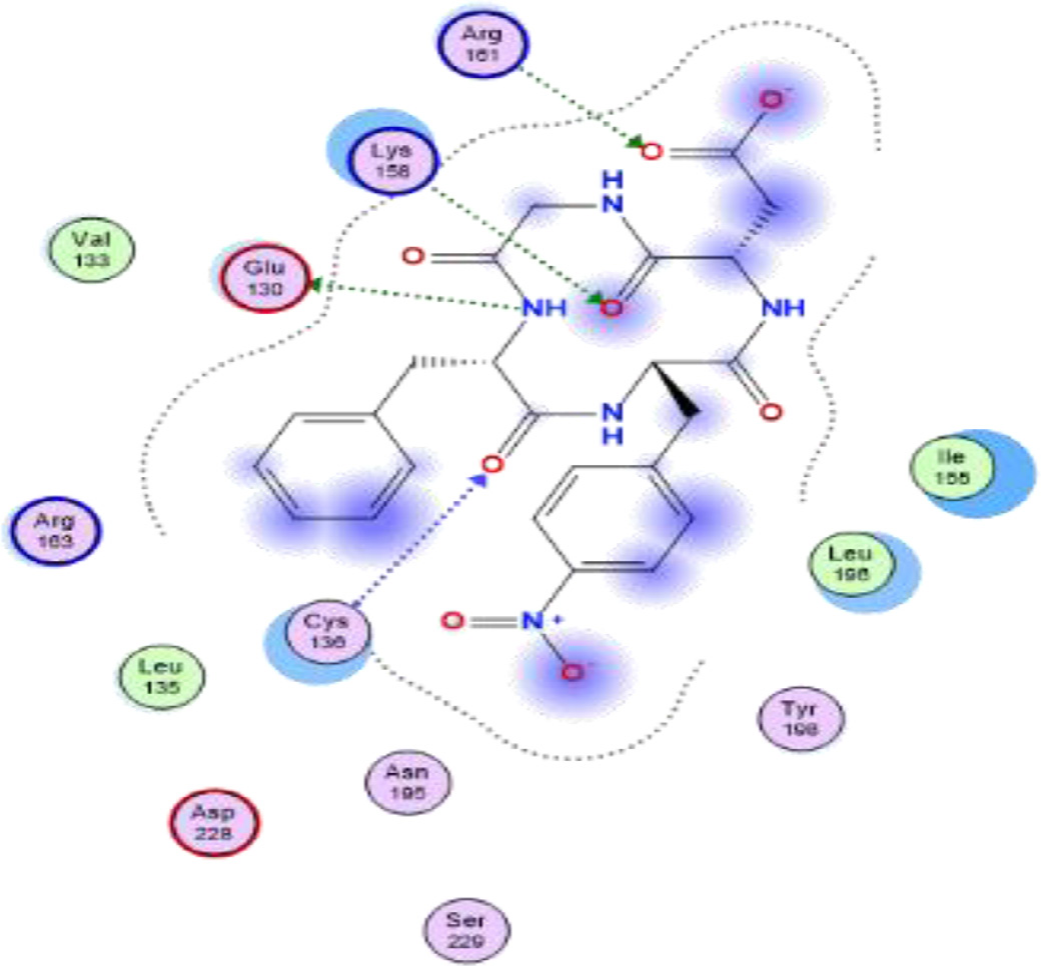
2-dimensional structure for docked Compound 6 against human Caspase 1 [pdb id: 6F6R].

**Fig. 7:**
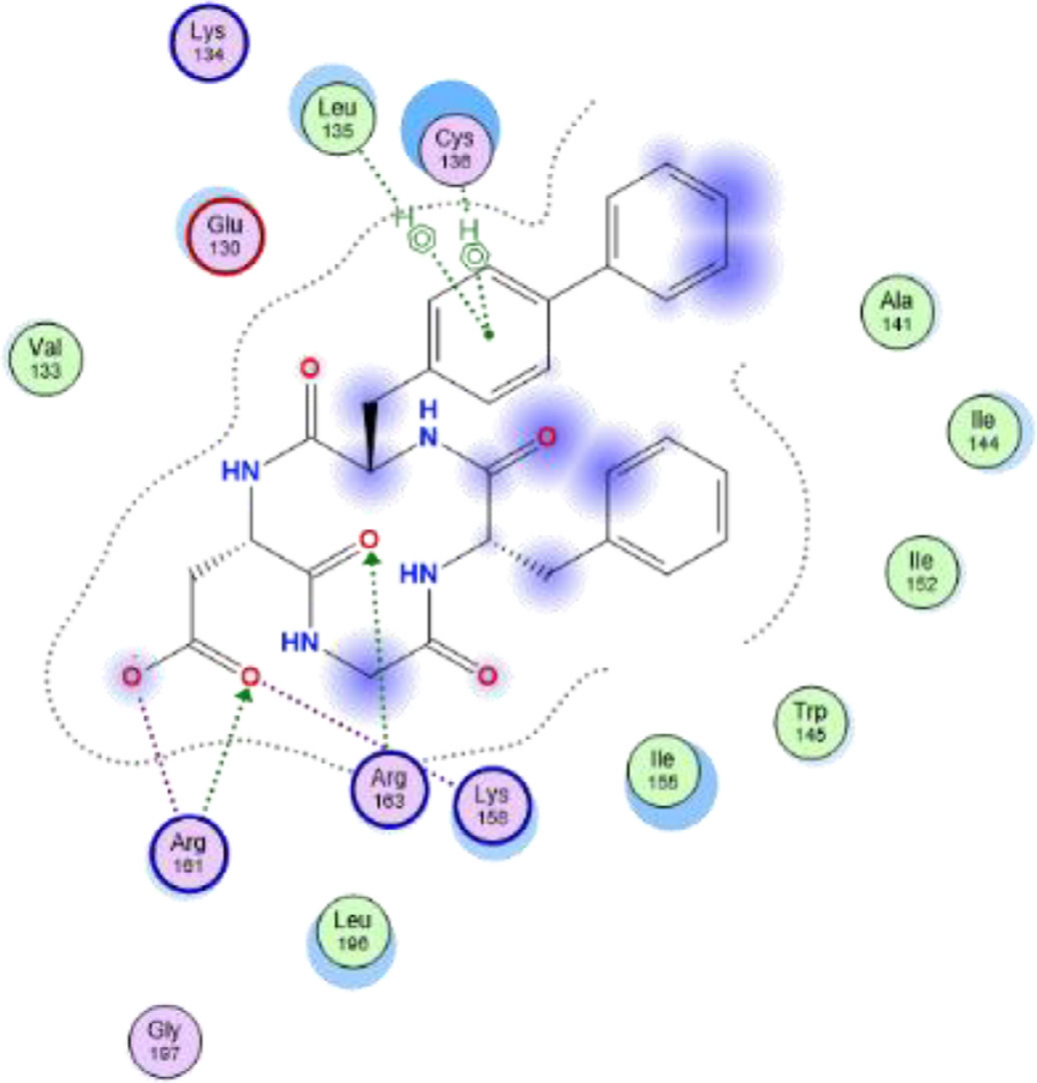
2-dimensional structure for docked Compound 7 against human Caspase 1 [pdb id: 6F6R].

**Fig. 8:**
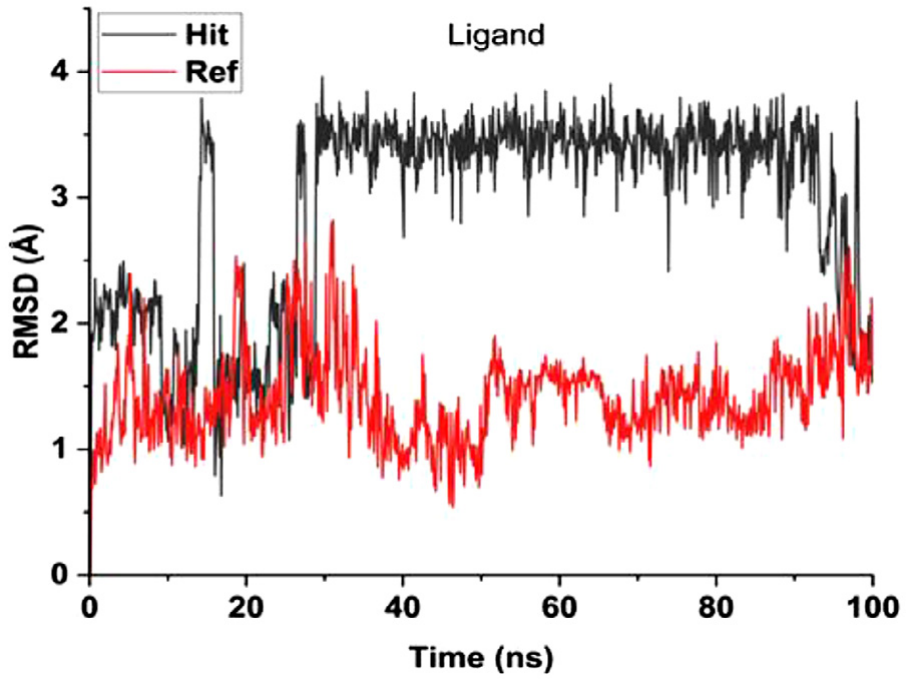
RMSD for compound 7 and VX-740 against human Caspase 1 (6F6R). Note: Hit: Compound 7, Ref: VX-740.

Table 7 reveals the ligand and amino acid residue involved in the interaction between the compounds and the human caspase 1 (6F6R). It also shows the distance between the ligand and the active site of the receptor. The two-dimensional structure scheme showing the interaction between each investigated compound and the proteins in the active site human caspase 1 receptor (6F6R) is displayed in Fig. 1, Fig. 2, Fig. 3, Fig. 4, Fig. 5, Fig. 6, Fig. 7–8.

**Table 5:** Predicted orbital energy levels for compounds optimized in ethanol.

**Table 6:** Calculated binding affinity for examined complexes.

	Scoring (kcal/mol)
1	-6.5045414
2	-6.9591408
3	-7.248467
4	-6.9861174
5	-7.0307384
6	-6.8699188
7	-7.4287872
VX-740	-6.7491722

**Table 7:** Predicted non bonding interaction for examined complexes.

	Ligand	Receptor	Interaction	Distance	E(Kcal/mol)
1	O2 2	NH2 ARG 161 (A)	H-acceptor	3.52	-0.7
	O13 13	N CYS 136 (A)	H-acceptor	3.37	-0.8
	O26 26	NH1 ARG 163 (A)	H-acceptor	3.07	-1.15
2	O24 24	NH1 ARG 163 (A)	H-acceptor	2.96	-3.6
	O24 24	NH2 ARG 163 (A)	H-acceptor	3.22	-1.7
	O26 26	N CYS 136 (A)	H-acceptor	2.94	-1.3
	O23 23	NH1 ARG 163 (A)	Ionic	3.01	-4.4
	O23 23	NH2 ARG 163 (A)	Ionic	3.8	-1
	O24 24	NH1 ARG 163 (A)	Ionic	2.96	-4.7
	O24 24	NH2 ARG 163 (A)	Ionic	3.22	-3.2
	6-ring	CE LYS 158	pi-H	4.48	-0.5
3	C28 28	O CYS 136 (A)	H-donor	3.46	-0.7
	O13 13	NH2 ARG 163 (A)	H-acceptor	3.16	-2.9
	O23 23	NH1 ARG 161 (A)	H-acceptor	2.96	-2.5
	O24 24	CD LYS 158 (A)	H-acceptor	3.02	-0.6
	O24 24	NH2 ARG 161 (A)	H-acceptor	2.96	-2.2
	O26 26	N CYS 136 (A)	H-acceptor	2.9	-1.6
	O23 23	NH1 ARG 161 (A)	Ionic	2.96	-4.8
	O23 23	NH2 ARG 161 (A)	Ionic	3.74	-1.1
	O24 24	NH1 ARG 161 (A)	Ionic	3.66	-1.3
	O24 24	NH2 ARG 161 (A)	Ionic	2.96	4.7
4	O18 18	N CYS 136 (A)	H-acceptor	2.94	-3.3
	026 26	NZ LYS 158 (A)	H-acceptor	2.54	-4.6
	6-ring	CG2 ILE 155 (A)	pi-H	3.77	-0.7
5	C20 20	OE2 GLU 130 (A)	H-donor	3	-1
	O13 13	NZ LYS 158 (A)	H-acceptor	3.22	-2.2
	O23 23	CE LYS 158 (A)	H-acceptor	3.51	-0.6
	O23 23	NH2 ARG 161 (A)	H-acceptor	2.78	-0.7
	O26 26	NH1 ARG 163 (A)	H-acceptor	3.47	-1.7
	O23 23	NH2 ARG 161 (A)	Ionic	2.78	-6.2
	O24 24	NH2 ARG 161 (A)	Ionic	2.65	-7.3
	O24 24	NH1 ARG 163 (A)	Ionic	3.25	-3
6	N3 3	OE2 GLU 130 (A)	H-donor	3.47	-0.6
	O2 2	N CYS 136 (A)	H-acceptor	3	-4.4
	O18 18	NZ LYS 158 (A)	H-acceptor	3.16	-6.2
	O23 23	NH1 ARG 161 (A)	H-acceptor	3.05	-9.4
	O23 23	NH1 ARG 161 (A)	Ionic	3.05	-4.1
	O23 23	NH2 ARG 161 (A)	Ionic	3.8	-1
7	O18 18	NH1 ARG 163 (A)	H-acceptor	3.19	-2.6
	O18 18	NH2 ARG 163 (A)	H-acceptor	3.26	-1.7
	O23 23	NH2 ARG 161 (A)	H-acceptor	3.12	-1.3
	O23 23	NZ LYS 158 (A)	Ionic	3.82	-0.9
	O23 23	NH2 ARG 161 (A)	Ionic	3.12	-3.7
	O24 24	NH2 ARG 161 (A)	Ionic	2.95	-4.8
	6-ring	CA LEU 135 (A)	pi-H	4.18	-0.8
	6-ring	N CYS 136 (A)	pi-H	3.62	-0.8

Table 8, Table 9 shows the result from the pharmacokinetic study of the compound with the best binding affinity (Compound 7) and the reference compound. The result was presented in ADMET-predicted profile classification and ADMET-predicted profile regression format. The predictions for the absorption model were Blood–Brain Barrier, Human Intestinal Absorption, Caco-2 Permeability, P-glycoprotein Substrate, P-glycoprotein Inhibitor and Renal Organic Cation Transporter. The distribution model is Subcellular localization, metabolism model was CYP450 2C9 Substrate, CYP450 2D6 Substrate, CYP450 3A4 Substrate, CYP450 1A2 Inhibitor, CYP450 2C9 Inhibitor, CYP450 2D6 Inhibitor, CYP450 2C19 Inhibitor, CYP450 3A4 Inhibitor and CYP Inhibitory Promiscuity and toxicity model: the Human Ether-a-go-go-Related Gene Inhibition, AMES Toxicity, Carcinogens, Fish Toxicity, Tetrahymena Pyriformis Toxicity, Honey Bee Toxicity, Biodegradation, Acute Oral Toxicity and Carcinogenicity (Three-class). For ADMET profiling prediction the following regression factors were considered aqueous solubility, Caco-2 Permeability (absorption) and Rat Acute Toxicity, Fish Toxicity and Tetrahymena Pyriformis Toxicity (toxicity).

**Table 8:** Predicted pharmacokinetic evaluation for compound 7.

*ADMET Predicted Profile — Classification*		
Model	Result	Probability
**Absorption**		
Blood-Brain Barrier	BBB-	0.6541
Human Intestinal Absorption	HIA±	0.8887
Caco-2 Permeability	Caco2-	0.7925
P-glycoprotein Substrate	Substrate	0.6391
P-glycoprotein Inhibitor	Non-inhibitor	0.9087
	Non-inhibitor	0.9682
Renal Organic Cation Transporter	Non-inhibitor	0.9231
**Distribution**		
Subcellular localization	Mitochondria	0.6841
**Metabolism**		
CYP450 2C9 Substrate	Non-substrate	0.8071
CYP450 2D6 Substrate	Non-substrate	0.8191
CYP450 3A4 Substrate	Non-substrate	0.6056
CYP450 1A2 Inhibitor	Non-inhibitor	0.8966
CYP450 2C9 Inhibitor	Non-inhibitor	0.9276
CYP450 2D6 Inhibitor	Non-inhibitor	0.9467
CYP450 2C19 Inhibitor	Non-inhibitor	0.9347
CYP450 3A4 Inhibitor	Non-inhibitor	0.9231
CYP Inhibitory Promiscuity	Low CYP Inhibitory Promiscuity	0.9536
**Excretion**		
**Toxicity**		
Human Ether-a-go-go-Related Gene Inhibition	Weak inhibitor	0.9903
	Non-inhibitor	0.8005
AMES Toxicity	Non AMES toxic	0.8748
Carcinogens	Non-carcinogens	0.9227
Fish Toxicity	High FHMT	0.9579
Tetrahymena Pyriformis Toxicity	High TPT	0.9027
Honey Bee Toxicity	Low HBT	0.8209
Biodegradation	Not ready biodegradable	0.9959
Acute Oral Toxicity	III	0.6314
Carcinogenicity (Three-class)	Non-required	0.7671
*ADMET Predicted Profile — Regression*		
**Model**	**Value**	**Unit**
**Absorption**		
Aqueous solubility	-3.0091	LogS
Caco-2 Permeability	-0.4886	LogPapp, cm/s
**Distribution**		
**Metabolism**		
**Excretion**		
**Toxicity**		
Rat Acute Toxicity	2.3315	LD50, mol/kg
Fish Toxicity	1.8950	pLC50, mg/L
Tetrahymena Pyriformis Toxicity	0.0777	pIGC50, ug/L

**Table 9:** Predicted pharmacokinetic evaluation for reference compound.

*ADMET Predicted Profile — Classification*		
Model	Result	Probability
**Absorption**		
Blood-Brain Barrier	BBB-	0.8386
Human Intestinal Absorption	HIA±	0.9821
Caco-2 Permeability	Caco2-	0.7237
P-glycoprotein Substrate	Substrate	0.7204
P-glycoprotein Inhibitor	Inhibitor	0.7443
	Non-inhibitor	0.9365
Renal Organic Cation Transporter	Non-inhibitor	0.7706
**Distribution**		
Subcellular localization	Mitochondria	0.5360
**Metabolism**		
CYP450 2C9 Substrate	Non-substrate	0.8340
CYP450 2D6 Substrate	Non-substrate	0.8350
CYP450 3A4 Substrate	Substrate	0.6186
CYP450 1A2 Inhibitor	Non-inhibitor	0.8814
CYP450 2C9 Inhibitor	Non-inhibitor	0.7453
CYP450 2D6 Inhibitor	Non-inhibitor	0.8095
CYP450 2C19 Inhibitor	Non-inhibitor	0.7977
CYP450 3A4 Inhibitor	Non-inhibitor	0.5643
CYP Inhibitory Promiscuity	High CYP Inhibitory Promiscuity	0.5861
**Excretion**		
**Toxicity**		
Human Ether-a-go-go-Related Gene Inhibition	Weak inhibitor	0.9197
	Non-inhibitor	0.5157
AMES Toxicity	Non AMES toxic	0.5288
Carcinogens	Non-carcinogens	0.8882
Fish Toxicity	High FHMT	0.9994
Tetrahymena Pyriformis Toxicity	High TPT	0.9617
Honey Bee Toxicity	Low HBT	0.8033
Biodegradation	Not ready biodegradable	1.0000
Acute Oral Toxicity	III	0.6686
Carcinogenicity (Three-class)	Non-required	0.6185
*ADMET Predicted Profile — Regression*		
**Model**	**Value**	**Unit**
**Absorption**		
Aqueous solubility	-3.2289	LogS
Caco-2 Permeability	0.3905	LogPapp, cm/s
**Distribution**		
**Metabolism**		
**Excretion**		
**Toxicity**		
Rat Acute Toxicity	2.4106	LD50, mol/kg
Fish Toxicity	1.3558	pLC50, mg/L
Tetrahymena Pyriformis Toxicity	0.4015	pIGC50, ug/L

**Fig. 9:**
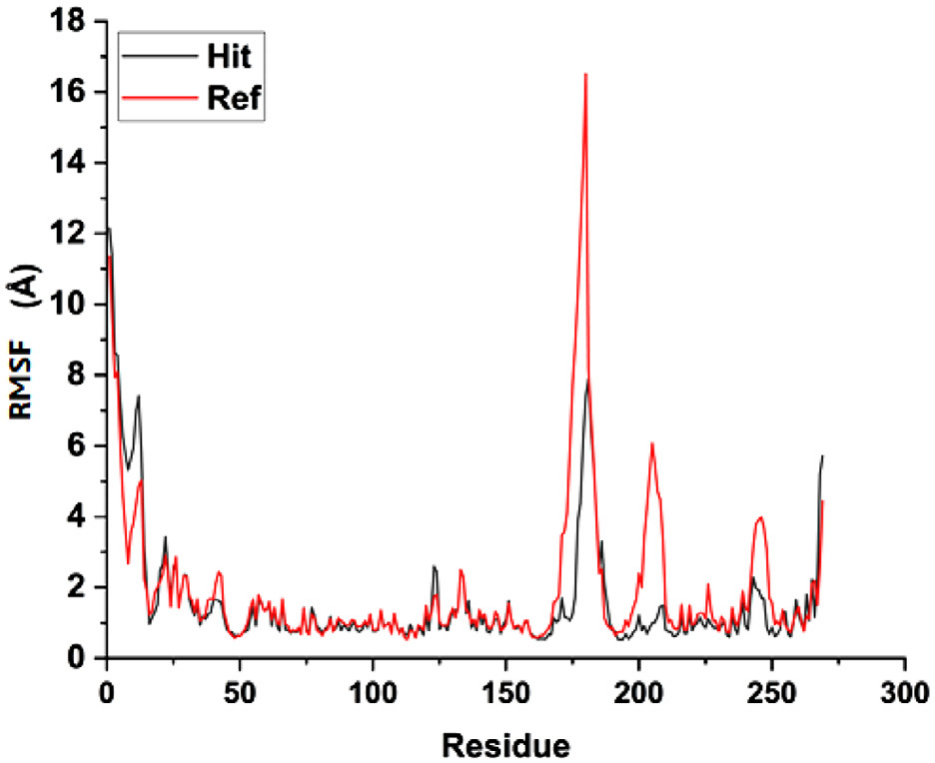
RMSF for compound 7 and VX-740 against human Caspase 1 (6F6R). Note: Hit: Compound 7, Ref: VX-740.

Table 10 showed the calculated binding energy between the lead compound and the reference compound. The free energy calculated in this work was ΔG_gas_ (gas phase energy), ΔG_solv_ (solvation free energy) and ΔG_bind_ (binding free energy). The calculated gas phase energy, solvation free energy and binding free energy for compound 7 were -37.4808 kcal/mol, 20.6774 kcal/mol and -16.8034 kcal/mol. More so, the gas phase energy, solvation free energy and binding free energy calculated for the reference compound were -61.5856 kcal/mol, 18.5007 kcal/mol and -15.0850 kcal/mol. Also, Fig. 8, Fig. 9 revealed the root mean square deviation (RMSD) carried out in 100ns and root mean square fluctuation (RMSF) respectively. The lead compound was represented in black line while the reference compound was represented in red line.

**Table 10:** Calculated binding energy for compound 7-6F6R and Ref-6F6R.

Complex	ΔG_gas_	ΔG_solv_	ΔG_bind_ (kcal/mol)
Hit	-37.4808	20.6774	-16.8034
Reference	-61.5856	18.5007	-15.0850

Note: Hit: Compound 7, Reference: Vx-740

## 4 Experimental Design, Materials and Methods

The 2-dimensional structures of the compounds under investigations were modelled using Chemdraw software and further subjected to Spartan 14 software which thereby convert the compounds to 3-dimensional structure. Spartan 14 software was used to optimize the Cylo phe-Ala-Asp-Gly compounds using density functional theory methodology via 6-31+ G* as basis set for the entire compounds which were optimized three phases: vacuum, water and ethanol. Optimization of the compounds in these three phases generated various molecular descriptors which describes the drug like activities of the compounds under investigation [1]. Also, human caspase 1 enzyme with pdb id: 6F6R [2] was retrieved from the protein data bank and the enzyme was treated using Molecular operating environment software to get rid of water molecules, and other small molecules before subjecting to docking calculation [[3], [4], [5]]. The small molecule retrieved from the receptor was docked into the active site of the receptor and the docked retrieved compound was placed on the original retrieved compound using discovery studio software to discern the level of deviation. It was observed that the RMSD was closer to 1 which confirmed the reliability of docking method used in this work. The examined enzyme structure was optimized using quickprep tool in order to repair and prepare the receptor before docking calculation. Human capase-1 enzyme was subjected to molecular operating environment to identify the potential binding sites the the Cylo phe-Ala-Asp-Gly compounds will bind with. Scoring of the calculated binding affinity measure in kcal/mol was also obtained and the result was accurately presented.

The interaction between compound 7 as well as the reference compound and the caspase-1 enzyme (PDB ID: 6F6R) for 100 ns were examined using molecular dynamics (MD).The simulated system was solvated with water molecules and the addition of counterions to neutralize the system. The CHARMM force field was typically chosen to model the interactions between the enzyme, compound, water, and ions. An initial energy minimization was performed to relieve any steric clashes, followed by equilibration in the NPT ensemble to stabilize temperature and pressure. The system was then subjected to a 100 ns production run, with periodic updates of atom positions and velocities using an integration time step of 1-2 fs. Throughout the simulation, key properties such as the binding stability, conformational changes of caspase-1, and interactions with compound 7 as well as the reference compound were monitored. The trajectory data were then analyzed to gain insights into binding affinities, molecular interactions, and dynamics of the enzyme-ligand complex [[6], [7], [8], [9]].

## Limitations

Not applicable

## Ethics Statement

This study does not involve studies with animals and humans*.*

## CRediT authorship contribution statement

**Faith Eniola Olujinmi:** Conceptualization, Methodology, Data curation, Writing – original draft, Visualization, Investigation, Writing – review & editing. **Chijioke John Ajaelu:** Conceptualization, Methodology. **Sunday Adewale Akintelu:** Data curation, Writing – original draft, Visualization, Investigation, Writing – review & editing. **Abel Kolawole Oyebamiji:** Conceptualization, Methodology, Data curation, Writing – original draft, Visualization, Investigation, Writing – review & editing.

## Data Availability

Mendeley DataDataset on Potential Biochemical Activities of Cylo phe-Ala-Asp-Gly-based Compounds as Caspase 1 Inhibitor (Original data).

## References

[1] Oyebamiji A.K., Akintelu S.A., Akintayo E.T., Akintayo C.O., Aworinde H.O., Adekunle O.D. (2023). Dataset on substituents effect on biological activities of linear RGD-containing peptides as potential anti-angiotensin converting enzyme. Data Br..

[2] Archana C.M., Harini K., Showmya J.J., Geetha N. (2014). An in-silico docking study of chromolaena odorata derived compounds against antimalarial activity. Int. J. Pharmacol. Sci. Bus. Manag..

[3] Oyebamiji A.K., Olujinmi F.E., Aworinde H.O., Oke D.G., Akintelu S.A., Akintayo E.T., Akintayo C.O., Babalola J.O. (2024). Dataset on anti-human insulin-degrading enzyme activities of cyclic tetra peptides: insight from in silico approach. Data Br..

[4] Attique S.A., Hassan M., Usman M., Atif R.M., Mahboob S., Al-Ghanim K.A., Bilal M., Nawaz M.Z. (2019). A molecular docking approach to evaluate the pharmacological properties of natural and synthetic treatment candidates for use against hypertension. Int. J. Environ. Res. Public Health.

[5] Mahmoud N.F., Abbas A.A., Mohamed G.G. (2021). Synthesis, characterization, antimicrobial, and MOE evaluation of nano 1, 2, 4-triazole-based Schiff base ligand with some d-block metal ions. Appl. Organomet. Chem..

[6] Wei H., McCammon J.A. (2024). Structure and dynamics in drug discovery. npj Drug Discov..

[7] Bhati S.K., Anjum F., Shamsi A. (2025). In silico screening and molecular dynamics analysis of natural DHPS enzyme inhibitors targeting *Acinetobacter baumannii*. Sci. Rep..

[8] Zhen H., Hu Y., Liu X., Fan G., Zhao S. (2024). The protease caspase-1: activation pathways and functions. Biochem. Biophys. Res. Commun..

[9] András F.W., Justin A.L. (2023). charmm2gmx: an automated method to port the CHARMM additive force field to GROMACS. J. Chem. Inf. Model..

